# Climate Change and Aging Health in Developing Countries

**DOI:** 10.1002/gch2.202200246

**Published:** 2023-08-11

**Authors:** Sabrina Maria Sarkar, Bablu Kumar Dhar, Mochammad Fahlevi, Selim Ahmed, Md. Jamal Hossain, Mohammad Meshbahur Rahman, Md. Abu Issa Gazi, Ranjithkumar Rajamani

**Affiliations:** ^1^ Young Women's Christian Association (YWCA) Chittagong 4000 Bangladesh; ^2^ Department of Business Administration Daffodil International University Dhaka Savar 1340 Bangladesh; ^3^ Business Administration Division Mahidol University International College Mahidol University Salaya 73170 Thailand; ^4^ Management Department BINUS Online Learning Bina Nusantara University Jakarta 11480 Indonesia; ^5^ World School of Business World University of Bangladesh Dhaka Dhaka 1230 Bangladesh; ^6^ Department of Pharmacy State University of Bangladesh 77 Satmasjid Road, Dhaka Dhanmondi 1205 Bangladesh; ^7^ Department of Biostatistics National Institute of Preventive and Social Medicine (NIPSOM) Dhaka 1212 Bangladesh; ^8^ School of Management Jiujiang University Jiujiang 332005 China; ^9^ Faculty of Health and Life Sciences INTI International University Persiaran Perdana BBN, Putra Nilai Nilai Negeri Sembilan 71800 Malaysia

**Keywords:** aging health, climate change, developing countries

## Abstract

The climate of the Earth has changed throughout history. Climate change negatively impacts human rights in a wide range of ways. The study aims to find out the impact of climate change on aging health in developing countries. The study found that public health will be devastated if climate change continues unabated. Countries that are least responsible for global warming are most susceptible to the effects of higher temperatures, such as death and disease. In low‐ and middle‐income countries, disasters are more likely to happen to people aged 60 and over. Although climate change affects all of us, older people are especially at risk from it, as evidenced by a growing body of research. The study also offers countermeasures and suggestions to develop aging health in developing countries affected by climate change.

## Graphical abstract

The primary focus of this study is to examine how climate change impacts the health of older individuals in developing nations. The research reveals that if climate change continues unchecked, it will severely affect public health. Although developing countries are less responsible for global warming, they are more vulnerable to the detrimental effects of rising temperatures, which contribute to higher mortality and disease rates. Furthermore, older adults in low– and middle‐income countries are at an increased risk of experiencing disasters. Numerous studies provide evidence supporting the idea that climate change poses significant risks to older populations. The study proposes various measures and recommendations to address these challenges to enhance the health and well‐being of ageing individuals in developing countries affected by climate change.

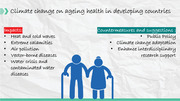

## 1 Introduction

In recent centuries, the Earth's surface has become warmer due to global warming. The climate of the Earth has changed throughout history. Since the last ice age ended 11 700 years ago, the planet has experienced seven cycles of glacial retreat and advancement, with the abrupt end of the last ice age marking the beginnings of the modern climate era, and civilization as well.^[^ ^1^ ^]^ As a result of increased carbon dioxide emissions into the atmosphere and other human activities, the planet's average surface temperature has risen ≈2.12 °F (1.18 °C) since the late 19th century.^[^ ^2^ ^]^ Most of the warming took place in the last 40 years, and the seven hottest years occurred in the last seven years. The hottest years on record are 2016 and 2020.^[^ ^3^ ^]^ Earth stores 90% of the extra energy in the ocean, which has absorbed much of the extra heat. The top 100 meters of the ocean have been warming by more than 0.6 °F (0.33 °C) since 1969.^[^ ^4^ ^]^ In the last century, the sea level rose by ≈8 inches (20 cm). In the last two decades, however, the growth rate has been nearly double what it was in the last century, and it has been accelerating every year.^[^ ^5^ ^]^

**Figure 1:**
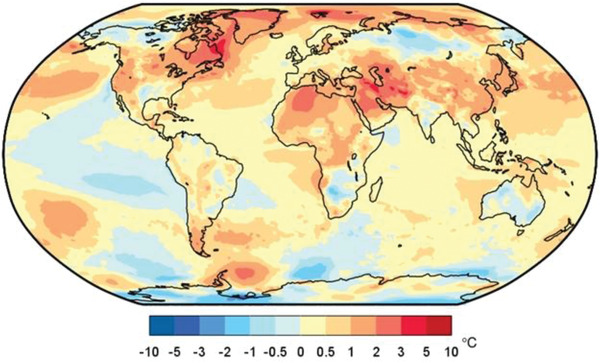
The mean global temperature in 2021.^[^ ^12^ ^]^

Temperatures worldwide have increased by 1.2 °C since pre‐industrial times. According to the World Meteorological Organization (WMO),^[^ ^6^ ^]^ **Figure** **1** shows that the mean global temperature in 2021 was ≈1.09 °C greater than the average of 1850–1900 (based on data from January to September). In June 2021, global surface temperature was 0.88 °C (1.58 °F) because of greenhouse gases (water vapor, CO_2_, CH_4_, and ozone).^[^ ^7^ ^]^ During the 21st century, global warming is expected to exceed 2 °C.^[^ ^8^ ^]^ It will be impossible to achieve the goals of the 2015 Paris Climate Agreement unless CO_2_ emissions and other greenhouse gases are rapidly reduced.

Climate change negatively impacts human rights in a wide range of ways. Public health will be devastated if climate change continues unabated. The world currently faces a climate emergency due to human‐caused global warming and pollution, which will cause a massive ecological and humanitarian disaster far larger than the current COVID‐19 pandemic.^[^ ^9^, ^10^ ^]^ In the past, the international climate agenda has been mostly focused on development. However, the concept has been evolving rapidly due to the growing evidence about its impact on public health worldwide. A number of factors contribute to these impacts, including geography, poverty, gender, age, nationality, birth status, and disabilities, which fall disproportionately on those who are already most vulnerable. Climate‐related diseases, heat stress, and sudden‐onset or slow‐onset disasters, including those that affect physical and mental health, are major threats to the ageing population, particularly older people with disabilities and older women.

Our ability to lower risks and be prepared for climate change by understanding the threat it poses to human health is crucial. Hence, the purpose of this article is to provide people with a better understanding of climate change and the need for specific interventions to support the adaptation of the older adult population in developing countries to the changing weather conditions.

## 2 Literature Review

**Figure 2:**
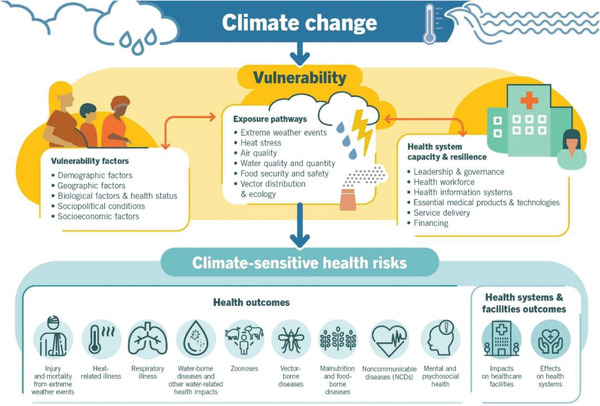
Climate‐sensitive health risks (Source: WHO, 2021).

Global warming will have an adverse impact on human health in addition to the visible effects on people's livelihoods. Developing countries that are least responsible for global warming are most susceptible to the effects of higher temperatures, such as death and disease. Globally, developing countries are defined by the World Bank^[^ ^11^ ^]^ as having lower incomes and economic development than developed countries and fewer resources for healthcare, education, and other basic services. It is common for developing countries to face social, economic, and environmental challenges, such as poverty, inadequate infrastructure, and poor environmental health. Developing countries have varying economic, social, and environmental conditions and varying susceptibilities to climate change's health impacts. For example, some countries such as China and India have experienced rapid economic growth and improvements in healthcare and education, while others in sub‐Saharan Africa and Southeast Asia continue to face significant levels of poverty and limited access to basic services. These differences in social and economic conditions can have significant impacts on the health consequences of climate change. For instance, the elderly population in low‐income developing countries may be particularly vulnerable to the health effects of extreme weather events, air pollution, and vector‐borne diseases due to poor health infrastructure, limited access to healthcare services, and other environmental risk factors. According to the World Health Organization (WHO) report^[^ ^12^ ^]^ the impact of climate change is expected to increase death rates by more than 250000 per year between 2030 and 2050 due to physical (injury, mortality, heat‐related, respiratory, water‐borne, zoonoses, vector‐borne, malnutrition, noncommunicable) and mental and psychosocial diseases (**Figure** **2**).

Therefore, it is crucial to consider the specific social, economic, and environmental conditions of developing countries when examining the health impacts of climate change. A section on developing countries could provide data on key characteristics such as income, education, infrastructure, and environmental health, which would then help to compare and contrast the health outcomes between developing and developed countries. This would aid in identifying the unique challenges and opportunities for adaptation and mitigation efforts in these contexts, and developing tailored interventions that are appropriate for the specific needs of different countries and populations.

Changing weather conditions are one factor that influences the spread of diseases transmitted by vectors (such as fleas, ticks, and mosquitoes), which spread pathogens.^[^ ^13^ ^]^ Geographic and seasonal patterns of vector populations, as well as their ability to transmit diseases, are significantly influenced by factors such as land use, socioeconomics, culture, pest control, health care access, and human response to disease risks.^[^ ^14^ ^]^ Sub‐Saharan Africa, the Indian oceans and the coasts of the Pacific are expected to be most affected by climate change. Human health conditions are predicted to become worse due to global warming, especially in tropical regions. The increase in temperature in Africa is accompanied by a rise in mosquito populations, which increases the risk for mosquito‐borne diseases like malaria and dengue.^[^ ^15^ ^]^

People with compromised health, such as the elderly and the sick, may suffer serious health effects when exposed to prolonged periods of high temperatures. Overall 80% of noncommunicable diseases are taking place in low‐ and middle‐income countries.^[^ ^16^ ^]^ Heart disease sufferers are more susceptible to increased temperatures since their cardiovascular systems must work harder to maintain body temperature. Increasing ozone levels and particulate matter levels due to climate change will affect human health in some locations. In addition to diminished lung function, increased hospital admissions and emergency room visits for asthma, and premature deaths, ground‐level ozone (which is a key component of smog) is linked to many other health issues. Temperatures over 90 °F can increase ozone levels, which damages lung tissue and causes respiratory complications, such as asthma attacks.^[^ ^17^, ^18^ ^]^

Waterborne illnesses are likely to increase with more frequent heavy rain, including those associated with sewage contamination of drinking water. By causing weather changes, high temperatures, fluctuating rainfall, and water shortages, climate change is a major cause of discomfort. In areas with poorly developed drainage systems, heavy rainfall can result in water stagnation increasing the risk of waterborne illnesses. A changing climate can put more strain on health systems. Disease development can be adversely affected by all the effects of climate change, including higher temperatures and heavy rainfall. These changes have a documented impact on food security and safety as well.^[^ ^19^ ^]^ The world's natural framework may be affected by environmental changes. Developing countries face a major problem with diarrheal disease.^[^ ^20^ ^]^ Temperatures in the air and water, precipitation patterns, extreme rain events, and the seasons all influence the transmission of diseases. Because of environmental change, developing countries like India are experiencing a wide range of diseases, including cholera, shigellosis, typhoid, and food poisoning.^[^ ^21^ ^]^

Droughts are common because of global warming, particularly in Africa. There is a worrying expectation that climate change will threaten food production, food quality, food prices, and food distribution systems worldwide.^[^ ^22^ ^]^ It is predicted that many crop yields will decrease as a result of changes in precipitation, severe weather events, and weeds and pests competing for plant resources. It is also predicted that livestock and fish production will decrease. The number of people without adequate water and food in Africa will rise by 75 million to 250 million by 2020 as crop productivity declines by as much as 50 percent.^[^ ^23^ ^]^ In Asia, 130 million people may also face food shortages due to rising temperatures.^[^ ^24^ ^]^

An ordinary fire may cause far more damage in a developing country than it would in a developed country.^[^ ^25^ ^]^ Many forests are becoming more vulnerable to wildfires as a result of climate change. Droughts in some areas may be associated with high temperatures for long periods of time, contributing to dry conditions and driving wildfires.^[^ ^26^ ^]^ Among the particles in wildfire smoke are particulate matter, carbon monoxide, nitrogen oxides, and volatile organic compounds which can seriously impact local air quality as well as the air quality of downwind areas.^[^ ^27^ ^]^ Land clearing and slash‐and‐burn agriculture are common practices in developing countries—and are important to their economies. These practices pose significant risks. Brazilian wildfires accounted for nearly 223 thousand outbreaks throughout 2020, by far the most in South America. Over 74 thousand fires broke out in Argentina that year, which was second in the region.^[^ ^28^ ^]^

Extreme weather events can impact mental health in several ways, and mental illness is one of the most common causes of suffering in developing countries.^[^ ^29^ ^]^ People with no history of mental illness are more likely to experience mental health problems after disasters, as are those at risk.^[^ ^30^ ^]^ These reactions can be short‐lived or long‐lasting. Additionally, some people with mental illnesses are particularly sensitive to heat. Climate change has the potential to impact depression and other mental illnesses, as suicide rates fluctuate with the weather.^[^ ^31^ ^]^ During heat waves, dementia can cause hospitalization and death. People with mental illnesses, especially those with schizophrenia, are at risk in hot weather since their medications may interfere with temperature regulation or can even cause hyperthermia. The mental health impacts of environmental degradation and displacement are also less understood, as is the anxiety and despair some might feel as a result of climate change awareness.^[^ ^32^ ^]^

## 3 Impact of Climate Change on Aging Health in Developing Countries

The impacts of climate change on the health of aging populations are substantial, both in developed and developing countries. In developed countries, older adults are predominantly vulnerable to the adverse health effects of climate change, including extreme heat, air pollution, and infectious diseases. These risks have been shown to be heightened among older adults in the United States, where exposure to extreme heat has been linked to amplified hospitalization and mortality rates.^[^ ^33^ ^]^ Similarly, air pollution has been associated with various negative health outcomes among older adults, such as respiratory and cardiovascular diseases.^[^ ^34^ ^]^

In developing countries, older adults face similar health risks worsened by climate change, but their vulnerability is often intensified due to inadequate healthcare infrastructure and limited access to healthcare services. For instance, older adults in low‐income countries in sub‐Saharan Africa and Southeast Asia are particularly susceptible to vector‐borne diseases like malaria and dengue fever, which are expected to become more frequent and severe as a result of climate change.^[^ ^12^ ^]^ Moreover, the health impacts of climate change in developing countries can exacerbate existing health disparities and socio‐economic inequalities. Older adults who live in poverty or lack access to clean water and sanitation are at an increased risk of waterborne diseases and malnutrition due to extreme weather events and agricultural changes.^[^ ^35^ ^]^

The effects of climate change on human health are evident in both direct impacts, such as heatwaves and extreme weather events, and indirect impacts, such as the increased susceptibility to infectious diseases and air pollution. The occurrence of diseases and chronic non‐communicable diseases has increased significantly, especially in developing countries, where the disease burden has shifted from primarily infectious diseases to chronic non‐communicable diseases like hypertension, heart disease, stroke, and cancer.^[^ ^36^ ^]^ Chronic non‐communicable diseases are the main cause of death for people over 60.^[^ ^37^ ^]^ Chronic non‐communicable diseases such as those of the respiratory and cardiovascular systems are also affected by climate change. Furthermore, the older people have relatively low resistance and are often afflicted by various disorders. Climate change has a significant impact on the health of the elderly. In summary, the elderly people are sensitive to climate change, and climate change is a major challenge to healthy ageing.

According to the report of United Nations,^[^ ^38^ ^]^ around the world, there will be 761 million people 65 years of age or older in 2023. The number of people worldwide is expected to more than double by 2050, reaching over 1.5 billion. In 2050, older persons will make up 16.0% of the global population, up from 9.3% in 2020. A quarter of the world's population is projected to be 65 or older by mid‐century.^[^ ^39^ ^]^

**Figure 3:**
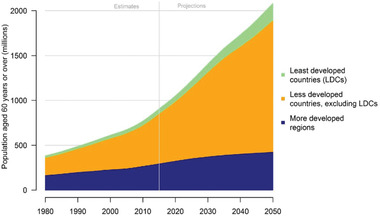
A comparison of the number of people over 60 from 1980 to 2050. Data Source: United Nations, Population Division.^[^ ^36^ ^]^

**Figure** **3** shows that developing regions have a much faster rate of aging population growth than developed regions. Because of this, the world's older population is growing in developing regions.^[^ ^38^ ^]^ Within low‐ and middle‐income countries, individuals aged 60 and above in particular exhibit a heightened likelihood of encountering disasters. While climate change affects the entire populace, the elderly possess specific susceptibilities, as substantiated by an expanding body of research.^[^ ^40^ ^]^ Moreover, older adults surpassing the age of 65 confront a notably increased peril of mortality amidst extreme weather occurrences due to their heightened vulnerability to temperature extremes.^[^ ^41^ ^]^ Their increased susceptibility to disease, reduced mobility, and the effects of food and water shortages makes them more vulnerable. The economic and social conditions of some older people also make them more vulnerable. They may be less able to cope with climate‐related stresses due to chronic health problems and social isolation.

The health impacts of climate change on ageing populations in developing countries are often more severe and complex than those in developed countries. While poverty and limited access to healthcare are key factors, the unique environmental and socio‐economic conditions of developing countries also play a significant role in exacerbating the risks faced by ageing populations.

### 3.1 Heat Waves and Cold Waves

There is a strong correlation between heat waves and cold waves and residents' health based on research on heat waves and cold waves.^[^ ^42^ ^]^ Cold waves commonly cause an increase in mortality and morbidity among people who suffer from cardiovascular diseases and cerebrovascular disorders. A heat wave increases blood circulation, dissipates heat, and loses water, ultimately increasing blood viscosity and electrolyte imbalance^[^ ^43^ ^]^ and a cold wave increases sympathetic nerve excitability, leading to increased heart rate, blood pressure, and left ventricular end‐diastolic pressure.^[^ ^45^ ^]^ The increase of cardiovascular system diseases is caused by the increase of myocardial oxygen consumption, the decrease of the ischemic threshold, the change in hemodynamics and the changes to the coagulation system.

The “China Cardiovascular Disease Report 2018” shows that cardiovascular diseases account for more than 40% of the deaths among urban residents, and rural areas rank higher than urban ones in terms of mortality rates. As a result of chronic noncommunicable diseases such as cardiovascular disease and cerebrovascular disease, mortality rates for the urban elderly in the age groups 60–64 and 65–69 are as high as 357.00/100 000 and 6.0997/100 000, respectively.^[^ ^46^, ^47^ ^]^ It is therefore imperative to reduce the risk of cardiovascular and cerebrovascular diseases among the elderly. Extreme temperatures both at home and abroad can cause an increase in cardiovascular disease and cerebrovascular disease morbidity and mortality. In tropical Vietnamese cities, heatwave events raise the overall risk of cardiovascular disease hospitalizations by 12.9%. Among the older people aged ≥65 years in Brisbane, Australia, the mortality rate of cardiovascular system disease increased by 3.7% when the temperature was 1 °C higher than the critical temperature, which is higher than 3.5% for the whole age group.^[^ ^48^ ^]^ According to Zeng et al.,^[^ ^49^ ^]^ in China the number of deaths due to cerebral hemorrhage during cold waves was 1.27 times that of non‐cold wave days, and on heat wave days it was 2.06 times that of non‐heat wave days.

The research of Zhang et al.^[^ ^50^ ^]^ and Raja et al.^[^ ^51^ ^]^ have found that heatwaves and greenness have deep impact on mortality among older adults in China and Bangladesh. According to the report of the World Health Organization,^[^ ^12^ ^]^ heatwaves have killed over 166000 people from 1998 to 2017 and most of them are older people from developing countries.^[^ ^52^, ^53^ ^]^ The relationship between heat and health outcomes is especially significant in cardiovascular diseases such as cardiovascular and cerebrovascular diseases, and the impact of cold waves usually shows a lag in their impact.^[^ ^54^ ^]^ For ageing people, heatwaves accelerate more blood circulation, increase heat dissipation and water loss, which, in turn, results in increased blood viscosity and electrolyte imbalance. In case of cold waves, sympathetic nerve excitability increases, increasing blood pressure, heart rate, left ventricular end‐diastolic pressure, myocardial oxygen consumption, and lowering the ischemia threshold, which increases disease incidence in the cardiovascular system of aging people.^[^ ^55^ ^]^

### 3.2 Extreme Calamities

Climate change‐induced extreme weather events, such as floods, droughts, and typhoons, will damage the living environment in many ways, including the health effects of water and insects as well as an increase in mortality and disability rates. Mental health is affected by disease incidence,^[^ ^56^ ^]^ and even mental health is affected by post‐traumatic stress disorder, anxiety.^[^ ^39^ ^]^ The body function of the older people declines with age, and the incidence of disability or semi‐disability increases as well.^[^ ^57^ ^]^ Increasing dependence on the external environment is necessary to maintain normal body function. As a result, when extreme climatic events occur, the elderly are forced to relocate because their living environment has been destroyed. Drinking water and food can be interrupted, resulting in malnutrition.^[^ ^58^ ^]^ Injury or death is more likely to occur in people who are vulnerable,^[^ ^59^ ^]^ such as those with limited mobility.

National Air Pollution–Morbidity and Mortality Effects Project has found that PM10 affects respiratory health, particularly pulmonary obstructive lung disease and pneumonia in the elderly over 65 years old.^[^ ^60^ ^]^ The older people who live in developed cities are more vulnerable to air pollution than those who live in less developed areas.^[^ ^61^ ^]^ Air pollution has adverse effects on respiratory diseases in different Chinese cities, according to research evidence. In Guangzhou, when the temperature is high, the interaction between PM10 and weather has a statistically significant effect on the death rate of respiratory diseases, especially in extreme cold weather.^[^ ^62^ ^]^ PM10 concentrations increase exponentially every 10 µg m^−3^, causing respiratory deaths to be 6.09% of non‐accidental events and cardiopulmonary deaths to be 3.36%.

There was no statistically significant difference in death rates from respiratory diseases between people under 65 years old and those aged 65 and over for every 10 µg m^−3^ increase in PM10 concentration. The older population over 65 years of age appears to be more sensitive to air pollution.^[^ ^63^ ^]^ The number of outpatient visits for respiratory diseases increased by 2.69%∼11.50% for every 10 µg m^−3^ increase in outdoor air pollutants such as PM2.5, PM10, SO_2_, and NO_2_, while the mortality rate of chronic respiratory diseases increased by 0.127–0.944%.^[^ ^64^ ^]^

A changing climate increases the frequency and intensity of extreme weather events such as flooding (from heavy rains, hurricanes, and coastal storms), droughts, and wildfires. Seniors are more likely to die in storms and floods. Over 2 million deaths and $3.64 trillion in losses were reported from these hazards, according to the World Health Organization,^[^ ^12^ ^]^ the death rate fell almost threefold between 1970 and 2019—from 50 000 deaths in the 1970s to less than 20 000 deaths in the 2010s—and >90% of those deaths occurred in developing and less developing countries.^[^ ^65^ ^]^ Older adults are at risk of both physical and mental health problems if they must evacuate during an extreme event. People with disabilities, chronic medical conditions, and those in nursing homes and assisted living facilities are among the most vulnerable.^[^ ^66^ ^]^ There is a possibility that interruptions in health care and problems associated with transporting patients with their medication, medical records, and any equipment such as oxygen could have a negative impact on their health. The power outages caused by extreme events can also affect electrically powered medical equipment and elevators, leaving some people unable to get treatment or evacuate.

### 3.3 Air Pollution

Polluted air contains a variety of complex compounds, such as pathogenic microorganisms and viruses, which can spread infectious diseases. As an example, the laboratory transmission of the Ebola virus has been confirmed by aerosol droplets,^[^ ^67^ ^]^ and the transmission of the SARS virus has also been confirmed by biological aerosol droplets.^[^ ^8^, ^56^, ^68^, ^69^ ^]^ As a result, it is not possible to ignore the risk of preventing the spread of viruses through aerosols and infecting older people with infectious diseases.

Acute respiratory infections and heart disease are caused by air pollution.^[^ ^70^ ^]^ Developing countries are more likely to record air pollution‐related deaths, as laws are weak or do not apply, vehicle emission standards are lower, and coal‐fired power plants are more common.^[^ ^71^, ^72^, ^73^ ^]^ In developing countries, air pollution is usually the most severe in the big cities because poor people live in informal settlements, often close to rubbish dumps.^[^ ^74^ ^]^ An example is Dandora, a huge smoldering dump site in Nairobi's eastern suburbs, which is surrounded by schools, churches, clinics, and shops. As temperatures rise, ground‐level ozone can be formed more easily and aeroallergens like ragweed pollen have a longer growing season. The growing number of wildfires and changing weather patterns contribute to increasing levels of pollution, dust, and smoke in the air.^[^ ^75^ ^]^ Nearly 2 million excess deaths in developing countries may be due to indoor air pollution, accounting for 4% of the global burden of diseases.^[^ ^76^ ^]^ Despite being healthy, older adults will be more likely to visit the emergency department and to be hospitalized. Chronic obstructive pulmonary disease and asthma, which occur in older adults, are worsened by poor air quality. Diabetes and obesity can also increase the risk of a heart attack in older adults due to air pollution.

### 3.4 Vector‐Borne Diseases

Every year there are more than 700 000 deaths from diseases such as malaria, dengue, schistosomiasis, human African trypanosomiasis, leishmaniasis, Chagas disease, yellow fever, Japanese encephalitis, and onchocerciasis.^[^ ^12^ ^]^ Ticks and mosquitoes will expand their ranges as a result of climate change and increased temperatures.^[^ ^77^ ^]^ As a result, people are more likely to be bitten by disease‐carrying ticks and mosquitoes. Among older adults, ticks are frequently reported to transmit Lyme disease.^[^ ^78^ ^]^ Elderly adults with weakened immune systems are at greater risk from West Nile and St. Louis encephalitis viruses, which are transmitted by mosquitoes.

### 3.5 Water Crisis and Contaminated Water Diseases

Drinking water and recreational water sources are at greater risk of contamination due to climate change. Lack of access to clean water and sanitation is found to be the cause of ≈80% of illnesses in developing countries. In both developed and many developing countries, water undergoes filtration and chlorination to remove disease‐causing organisms, thereby guaranteeing its suitability for drinking. This measure is essential not only in more advanced nations but also in numerous developing regions, where it plays a critical role in promoting the health and well‐being of a substantial portion, even a significant majority, of the population. Therefore, in developed countries, most of these diseases are not found. The developing world is still plagued by diseases like typhoid fever, cholera, and many others.^[^ ^79^ ^]^ A contaminated water supply poses a high risk of gastrointestinal illnesses for older adults. Health problems, including death, are more likely to affect those already ill. More than 28% of adults 75 years of age and older reported fair or poor health in 2013, compared with 6% of those 18–44 years of age.^[^ ^12^ ^]^

## 4 Countermeasures and Suggestions to Develop Aging Health in Developing Countries Due to Climate Change

### 4.1 Public Policy

Governments of developing countries should focus on their Conference on Ecological Environmental Protection when developing a national strategy to combat climate change.^[^ ^80^ ^]^ As a result, a strategy that actively addresses climate change will be given a higher ranking in the national strategy. Climate change, a global problem of public health, is affecting human health adversely by increasing the incidence and mortality of different communicable and non‐communicable diseases.^[^ ^81^ ^]^ Poor adaptation systems to climate change are a major problem in many developed and developing countries. Climate change is certain to affect the older people, and they are a vulnerable group.^[^ ^82^ ^]^ To promote healthy aging, the older people health service system must be improved, climate change must be integrated into the system, and health departments must take a more active role. A close collaboration between disease control and other departments is necessary.

### 4.2 Enhance Interdisciplinary Research Support

The health impacts of climate change on the elderly are not a key area of concern, even though the older people are sensitive to climate change. Researchers have described the direct and indirect effects of climate change on the health of the elderly population, while quantitative research has mainly examined the relationship between climate change and climate‐sensitive diseases and other health outcomes.^[^ ^41^ ^]^ There is a lack of research into the relationship between the older people and climate change and health response,^[^ ^83^ ^]^ the pathogenesis of climate change and common diseases in the elderly, and health economic evaluations of climate change‐related diseases. Research on the effects of population health is incomplete and unsystematic.^[^ ^84^ ^]^ For a better understanding of how climate change affects the health of older populations, more support is needed for research. It is essential to strengthen interdisciplinary research and bring into play the synergy of different professional fields so that the impact of climate change on the health of the elderly can be clarified. This involves geriatrics, the sociology of populations, meteorology, and epidemiology. In order to reduce the adverse effects of climate change on the health of the elderly and promote healthy ageing, we must understand why and how it impacts the health of the elderly.

### 4.3 Climate Change Adaptation for the Elderly

There is insufficient publicity and awareness of climate change in developing countries at present.^[^ ^85^, ^86^ ^]^ A recommendation is to incorporate the impact of climate change on health into health literacy. Actively publicize and popularize the impact of climate change on human health by popularizing health knowledge and promoting the health of older people. Taking health consultations, public lectures, or publicity slogans, brochures, and even exhibitions are excellent forms of health education and publicity, and pushing vigorously publicity activities on the impact of climate change on the health of older people is necessary.^[^ ^87^ ^]^ To improve elderly awareness of the health impacts of climate change, the developing countries should introduce major strategies, guidelines, and policies in fighting climate change as well as the impact of climate change on the health of the older people. Also, it is important to cultivate a group of propagandists, such as community workers closely associated with the elderly, nursing staff in nursing homes, and medical staff in medical institutions, to inform the older people about climate change and its adverse effects, as well as strengthening them through active intervention.

## 5 Conclusions

The elderly are greatly affected by climate change, resulting in wide‐ranging implications. The convergence of susceptible geographic areas and limited resilience to severe weather events and rising sea levels implies that individuals living in poverty within developing countries will experience the harshest repercussions. As a result of climate change, everyone will be affected, but the poor in particular will lose everything they have. The impact of climate change on the health of older adults, their families, and caregivers must be understood so that preventative measures can be taken. Societies with greater wealth have access to more advanced technological advancements that can decrease heat retention. The lack of technical knowledge, as well as resources and public health system, has made outbreak prevention difficult in developing countries. While the systematic search process was designed to minimize the risk of bias, there may be some limitations to this review. For example, the search was limited to studies published in English, which may have resulted in the exclusion of relevant studies conducted in other languages. Additionally, the inclusion criteria may have excluded studies that investigated other health impacts of climate change in developing countries.

## 6 Experimental Section

The research team identified the issues of vector‐borne diseases, wildfires, and extreme weather events as potential health impacts of climate change in developing countries based on a review of existing literature on the topic. These issues have been identified in various reports and studies as key health risks associated with climate change in developing countries. A systematic search process was utilized to locate relevant sources of information. The research team conducted searches across multiple academic databases, such as PubMed, Scopus, and Web of Science, to find articles published from 2010 to 2021. The search terms employed included “climate change,” “health,” “vector‐borne diseases,” “wildfires,” and “extreme weather events.” The scope of the search was limited to studies led in developing countries. Primarily, the team screened the titles and abstracts of the identified articles to determine their relevance to the research question. The inclusion criteria consisted of studies that scrutinized the link between climate change and health impacts in developing countries, with a particular focus on vector‐borne diseases, wildfires, and extreme weather events as potential health consequences. Articles meeting these criteria were then investigated to assess the extent to which they supported the research question. The research team assessed the quality of the articles based on established criteria for evaluating scientific literature, including study design, sample size, and statistical methods employed. Additionally, the team considered the consistency of results across different studies. By synthesizing the selected articles, a comprehensive overview of the health impacts of climate change in developing countries was provided, with specific attention given to vector‐borne diseases, wildfires, and extreme weather events. The results were compared and contrasted to identify common themes and patterns.

## Conflict of Interest

The authors declare no conflict of interest.

## Data Availability

Research data are not shared.

## References

[1] D. J. Meltzer , First Peoples in a New World: Populating Ice Age America, Cambridge University Press, Cambridge, MA 2021.

[2] F. Wu , Q. You , Z. Zhang , L. Zhang , Int. J. Climatol. 2020, 41.

[3] E. J. Rohling , M. Brown , H. Eakin , J. Eom , A. S. von der Heydt , Oxford Open Clim. Change 2021, 1, 1.

[4] https://www.lancetcountdown.org/our‐science/ (accessed: May 2023).

[5] R. S. Nerem , B. D. Beckley , J. T. Fasullo , B. D. Hamlington , D. Masters , G. T. Mitchum , Proc. Natl. Acad. Sci. USA 2018, 115, 2022. 29440401 10.1073/pnas.1717312115 PMC5834701

[6] https://public.wmo.int/en/media/press‐release/state‐of‐climate‐2021‐extreme‐events‐and‐major‐impacts (accessed: May 2023).

[7] https://climate.nasa.gov/resources/global‐warming‐vs‐climate‐change (accessed: April 2023.

[8] H. C. Ossebaard , P. Lachman , Int. J. Qual. Hlth. Care 2020, 33.

[9] F. K. Ayittey , B. K. Dhar , G. Anani , N. B. Chiwero , Hlth. Care Women Int. 2020, 41, 1210. 10.1080/07399332.2020.1809664 33616506

[10] A. Di Ciaula , M. Krawczyk , K. J. Filipiak , A. Geier , L. Bonfrate , P. Portincasa , Eur. J. Clin. Invest. 2021, 51. 10.1111/eci.13682 PMC8646618 34551123

[11] https://datahelpdesk.worldbank.org/knowledgebase/articles/906519‐world‐bank‐country‐and‐lending‐groups (accessed: May 2023).

[12] https://www.who.int/news‐room/fact‐sheets/detail/climate‐change‐and‐health (accessed: April 2023).

[13] J. Rocklöv , R. Dubrow , Nat. Immunol. 2020, 21, 479. 32313242 10.1038/s41590-020-0648-y PMC7223823

[14] A. L. Wilson , O. Courtenay , L. A. Kelly‐Hope , T. W. Scott , W. Takken , S. J. Torr , S. W. Lindsay , PLoS Neglect. Trop. Dis. 2020, 14, e0007831. 10.1371/journal.pntd.0007831 PMC6964823 31945061

[15] E. A. Mordecai , S. J. Ryan , J. M. Caldwell , M. M. Shah , A. D. LaBeaud , Lancet Pl. Health 2020, 4, e416. 10.1016/S2542-5196(20)30178-9 PMC7490804 32918887

[16] H. A. Rother , Sc. Total Env. 2020, 722, 137772. 32199361 10.1016/j.scitotenv.2020.137772

[17] G. D'Amato , H. J. Chong‐Neto , O. P. Monge Ortega , C. Vitale , I. Ansotegui , N. Rosario , I. Annesi‐Maesano , Allergy 2020, 75, 2219. 32589303 10.1111/all.14476

[18] M. Joshi , H. Goraya , A. Joshi , T. Bartter , Curr. Opinion Pulm. Med. 2020, 26, 119. 10.1097/MCP.0000000000000656 31851023

[19] J. Brubacher , D. M. Allen , S. J. Déry , M. W. Parkes , B. Chhetri , S. Mak , T. K. Takaro , Sci. Total Env. 2020, 728, 138808. 32570317 10.1016/j.scitotenv.2020.138808

[20] N. Mutono , J. A. Wright , H. Mutembei , J. Muema , M. L. Thomas , M. Mutunga , S. M. Thumbi , AAS Open Res 2021, 4. 34368620 10.12688/aasopenres.13225.1 PMC8311817

[21] M. Anas , M. A. Sami , Z. Siddiqui , K. Khatoon , M. T. Zeyad , A. Malik , in Microbiomes and the Global Climate Change, Springer, Singapore 2021.

[22] M. M. Blakstad , E. R. Smith , Lancet Pl. Health 2020, 4, e547. 10.1016/S2542-5196(20)30246-1 33278369

[23] A. Bhopal , H. Medhin , K. Bærøe , O. F. Norheim , Wor. Med. Health Pol. 2021, 13, 293.

[24] A. Bisht , M. P. Kamble , P. Choudhary , K. Chaturvedi , G. Kohli , V. K. Juneja , N. K. Taneja , Food Cont. 2021, 121, 107630.

[25] X. Zong , X. Tian , Y. Yin , Forests 2020, 11, 802.

[26] N. B. Arriagada , D. M. Bowman , A. J. Palmer , F. H. Johnston , in Extreme Weather Events and Human Health, Springer, Cham 2020.

[27] R. Xu , P. Yu , M. J. Abramson , F. H. Johnston , J. M. Samet , M. L. Bell , Y. Guo , New Eng. J. Med. 2020, 383, 2173. 33034960 10.1056/NEJMsr2028985

[28] P. Brando , M. Macedo , D. Silvério , L. Rattis , L. Paolucci , A. Alencar , C. Amorim , Flora 2020, 268, 151609.

[29] T. Clark , T. R. Zolnikov , Palgrave Handbook Clim. Resilient Societies, vol. 1, Palgrave Macmillan, Cham. 2020, pp. 1–26.

[30] M. Hrabok , A. Delorme , V. I. Agyapong , J. Anxiety Disord. 2020, 76, 102295. 32896782 10.1016/j.janxdis.2020.102295

[31] P. Cianconi , S. Betrò , L. Janiri , Front. Psychiat. 2020, 11, 74. 10.3389/fpsyt.2020.00074 PMC7068211 32210846

[32] L. A. Palinkas , M. Wong , Curr. Opin. Psychol. 2020, 32, 12. 31349129 10.1016/j.copsyc.2019.06.023

[33] R. Basu , Epidemiol. 2019, 30, 356.

[34] A. J. Cohen , M. Brauer , R. Burnett , H. R. Anderson , J. Frostad , K. Estep , M. H. Forouzanfar , Lancet 2017, 389, 1907. 28408086 10.1016/S0140-6736(17)30505-6 PMC5439030

[35] S. S. Myers , M. R. Smith , S. Guth , C. D. Golden , B. Vaitla , N. D. Mueller , et al., Annu. Rev. Publ. Health 2017, 38, 259. 10.1146/annurev-publhealth-031816-044356 28125383

[36] T. Zhou , H. Guan , J. Yao , X. Xiong , A. Ma , Qual. of Lif. Res. 2018, 27, 2799. 10.1007/s11136-018-1928-y PMC6208588 29980994

[37] M. K. Al‐Hanawi , Int. J. Equity Health 2021, 20, 1. 34321000 10.1186/s12939-021-01510-6 PMC8320210

[38] www.un.org/development/desa/pd/data/living‐arrangements‐older‐persons (accessed: April 2023).

[39] S. M. Sarkar , B. K. Dhar , S. S. Crowley , F. K. Ayittey , M. A. I. Gazi , Age. Int. 2021, 48, 222. 10.1007/s12126-021-09467-1 PMC8503868 34658464

[40] L. Hyde‐Smith , Z. Zhan , K. Roelich , A. Mdee , B. Evans , Env. Health Pers. 2022, 130, 1. 10.1021/acs.est.1c07424 PMC9069703 35412814

[41] L. Ayalon , N. Keating , K. Pillemer , K. Rabheru , Am. J. Geriat. Psychi. 2021, 29, 1038. 10.1016/j.jagp.2021.06.015 34294541

[42] M. Pascal , V. Wagner , A. Alari , M. Corso , A. L.e Tertre , Atmosph. Env. 2021, 249, 118249.

[43] K. L. Ebi , A. Capon , P. Berry , C. Broderick , R. de Dear , G. Havenith , O. Jay , Lancet 2021, 398, 698. 34419205 10.1016/S0140-6736(21)01208-3

[44] J. Moon , Env. Res. 2021, 195, 110762. 33515577 10.1016/j.envres.2021.110762

[45] A. J. Van Der Walt , J. M. Fitchett , Int. J. Climat. 2021, 41, 2060.

[46] C. Shi , L. Wang , J. Ye , Z. Gu , S. Wang , J. Xia , Y. Xie , Q. Li , R. Xu , N. Lin , BMC Infect Dis. 2021, 21, 1. 34238232 10.1186/s12879-021-06369-0 PMC8264491

[47] G. Zhai , K. Zhang , G. Chai , Air Qual., Atmosph. Health 2021, 14, 181.

[48] B. Xia , L. Buys , T. Yigitcanlar , Sus. 2021, 13, 9853.

[49] W. Zeng , M. Yu , W. Mai , M. Zhou , C. Zhou , Y. Xiao , W. Ma , Env. Res. 2022, 203, 111834. 34358501 10.1016/j.envres.2021.111834

[50] H. Zhang , L. Liu , Y. Zeng , M. Liu , J. Bi , J. S. Ji , Env. Pollut. 2021, 290, 118009. 34523521 10.1016/j.envpol.2021.118009

[51] R. Raja , M. S. N. Hredoy , M. K. Islam , K. A. Islam , M. S. G. Adnan , Env. Challenges 2021, 4, 100122.

[52] D. P. Bitencourt , M. V. Fuentes , A. E. Franke , R. B. Silveira , M. P. Alves , Int. J. Climatol. 2020, 40, 2464.

[53] A. J. van Der Walt , J. M. Fitchett , Int. J. Climatol. 2021, 41, 2060.

[54] T. Fritze , Sus. 2020, 12, 3664.

[55] J. Chambers , C. Chan 2020, 163, 539.

[56] B. K. Dhar , F. K. Ayittey , S. M. Sarkar , Glob. Challen. 2020, 4, 2000038. 10.1002/gch2.202000038 PMC7537036 33042575

[57] P. Zaninotto , G. D. Batty , M. Allerhand , I. J. Deary , J Epidemiol. Commun. Health 2018, 72, 685. 10.1136/jech-2017-210116 PMC6204948 29691286

[58] A. Donnelly , Nurs. Stand. 2018, 33, 69. 10.7748/ns.2018.e11169 29873473

[59] S. J. Curry , A. H. Krist , D. K. Owens , M. J. Barry , J. B. Wong , JAMA, J. Am. Med. Assoc. 2018, 320, 1678.

[60] S. Sanyal , T. Rochereau , C. N. Maesano , L. Com‐Ruelle , I. Annesi‐Maesano , Int. J. Env. Res. Pub. Health 2018, 15, 2487. 30412999 10.3390/ijerph15112487 PMC6266056

[61] C. Zhou , S. Li , S. Wang , Int. J. Env. Res. Publ. Health 2018, 15, 1565.

[62] N. Wang , K. Mengersen , S. Tong , M. Kimlin , M. Zhou , L. Wang , W. Hu , Env. Res. 2019, 179, 108748. 31561053 10.1016/j.envres.2019.108748

[63] T. M. Mata , F. Felgueiras , A. A. Martins , H. Monteiro , M. P. Ferraz , G. M. Oliveira , G. V. Silva , Env. 2022, 9, 86.

[64] X. Yang , N. Li , H. Mu , M. Ahmad , X. Meng , Gondwana Res 2022, 106, 303.

[65] J. Marshall , J. Wiltshire , J. Delva , T. Bello , A. J. Masys , in Global Health Security, (Eds: A. J. Masys , R. Izurieta , M. R. Ortiz ), Springer, Cham 2020, p. 143.

[66] E. Frankenberg , C. Sumantri , D. Thomas , Nat. Sus. 2020, 3, 614. 10.1038/s41893-020-0536-3 PMC7935047 33681474

[67] M. A. Schuit , J. Taylor , R. Dunning , D. Miller , D. Freeburger , L. Faisca , P. A. Dabisch , Aerosol Sci. Tech. 2021, 55, 458.

[68] F. K. Ayittey , N. B. Chiwero , B. K. Dhar , E. L. Tettey , Int. J. Clinic. Prac. 2021, 75, e15012. 10.1111/ijcp.15012 PMC9011565 34806816

[69] A. A. Rabaan , S. H. Al‐Ahmed , M. Al‐Malkey , R. Alsubki , S. Ezzikouri , F. H. Al‐Hababi , A. J. Rodriguez‐Morales , Infez. Med. 2021, 29, 10. 33664169

[70] K. Kirwa , C. M. Eckert , S. Vedal , A. Hajat , J. D. Kaufman , BMJ Open Resp. Res. 2021, 8, e000866. 10.1136/bmjresp-2020-000866 PMC7934778 33664125

[71] E. K. Chowdhury , B. K. Dhar , M. A. I. Gazi , J. Knowl. Econ. 2023, 14, 382.

[72] B. K. Dhar , I. Harymawan , S. M. Sarkar , Corp. Soci. Respon. Env. Manage. 2022, 29, 701.

[73] B. K. Dhar , S. M. Sarkar , F. K. Ayittey , Corp. Soci. Respon. Env. Manage. 2021, 29, 71.

[74] P. DeSouza , Air Qual. Atmosp. Health 2020, 13, 1487.

[75] Y. Luo , Y. Zhong , L. Pang , Y. Zhao , R. Liang , X. Zheng , Sci. Tot. Env. 2021, 754, 142460. 10.1016/j.scitotenv.2020.142460 33254849

[76] K. Burkart , K. Causey , A. J. Cohen , S. S. Wozniak , D. D. Salvi , C. Abbafati , V. Nangia , Lancet Planet. Health 2022,6, e586.35809588

[77] V. Mulderij‐Jansen , I. Gerstenbluth , A. Duits , A. Tami , A. Bailey , Paras. Vec. 2021, 14, 1. 10.1186/s13071-021-05011-x PMC8474927 34565464

[78] V. R. N. Cruvinel , T. R. Zolnikov , M. T. Obara , V. T. L. de Oliveira , E. M. Vianna , E. N. , F. S. G. do Santos , J. A. Scott , Wast. Manage. 2020, 105, 223. 10.1016/j.wasman.2020.02.001 32087540

[79] H. Fan , Y. Wang , Y. Wang , P. C. Coyte , Env. Sci. Pollut. Res. 2021, 29, 4219. 10.1007/s11356-021-15832-z 34403062

[80] H. M. Rahman , G. M. Hickey , Front. Env. Sc. 2019, 7, 2.

[81] T. Palmer , B. Stevens , Acad. Sci. 2019, 116, 24390. 10.1073/pnas.1906691116 PMC6900733 31792170

[82] M. J. Hornsey , K. S. Fielding , Pol. Rev. 2020, 14, 3.

[83] D. Schwela , in, Encyclopedia of Gerontology and Population Aging, Springer International Publishing, Cham 2021.

[84] M. Carroll , J. Walker , in Rural Gerontology (Eds: M. Skinner , R. Winterton , K. Walsh ), Routledge, Oxfordshire, UK 2021.

[85] J. Kotcher , E. Maibach , J. Miller , E. Campbell , L. Alqodmani , M. Maiero , A. Wyns , The Lancet Pl. Health 2021, 5, e316. 10.1016/S2542-5196(21)00053-X PMC8099728 33838130

[86] C. Singh , M. Madhavan , J. Arvind , A. Bazaz , Urban Clim. 2021, 36, 100783.

[87] R. S. Rodriguez , D. Ürge‐Vorsatz , A. S. Barau , Nat. Clim.Chan. 2018, 8, 181.

